# Social Anxiety Predicts Enhanced Electrocortical Sensitivity to the Mental State Meanings Revealed by Gaze Patterns

**DOI:** 10.1111/psyp.70409

**Published:** 2026-09-28

**Authors:** Irene Melani, Martha K. Berg, James Glazer, Shinobu Kitayama

**Affiliations:** ^1^ Department of Psychology University of Michigan Ann Arbor Michigan USA; ^2^ Department of Psychology Northwestern University Evanston Illinois USA

**Keywords:** event‐related potential, mental state inference, N400, semantic integration, social anxiety

## Abstract

Social anxiety—a fear of social interaction and negative evaluation—may be accompanied by heightened vigilance toward others' mental states, particularly those conveyed through gaze, as they provide cues about how one is being evaluated. We thus investigated whether individual differences in social anxiety predict neural sensitivity to the mental‐state meanings conveyed by gaze patterns. Fifty‐seven European American young adults varying in social anxiety viewed different gaze patterns, immediately followed by a mental‐state word that was either congruent or incongruent with the culturally recognized meaning of the gaze, while their electroencephalogram (EEG) was monitored. Our focus was N400—an event‐related potential (ERP) component indexing semantic expectation violations. We found the N400 amplitude to be more negative for incongruent (vs. congruent) mental‐state words. Notably, this N400 incongruity effect increased significantly with social anxiety. Moreover, the social anxiety effect was specific to mental state inference and was not observed in a comparable N400 incongruity effect involving animal stereotypes (*n* = 50). These findings suggest that social anxiety may confer enhanced neural sensitivity during the inference of others' mental states from gaze, even in the absence of overt performance differences. We discuss the implications of these findings with respect to the potential functional benefits of social anxiety.

## Impact Statement

Social anxiety entails apprehension about how one is socially evaluated and, as such, may heighten neural sensitivity during the inference of others' mental states from gaze. Here, we show that higher social anxiety predicts a more pronounced N400 response to mental‐state words that are incongruent (vs. congruent) with preceding gaze patterns. These results highlight a previously underappreciated aspect of social anxiety as potentially functional in detecting and coping with socially costly interactions.

## 1 Introduction

Social anxiety—a fear of social interactions linked to evaluation apprehension (Schlenker and Leary 1982)—has widely been considered detrimental (Hofmann 2007; Mellings and Alden 2000; Nolen‐Hoeksema 2000). Accordingly, it is often associated with impairments in social functioning and well‐being, making it difficult to identify circumstances under which social anxiety might confer any advantage. Nevertheless, it has been argued that psychological traits, including aversive states, that persist in the population may confer context‐dependent functional advantages despite their subjective costs (Keller and Nesse 2006; Nesse 2005). One domain in which such functions may be especially relevant is the interpretation of social cues that reveal others' mental states, providing insight into how one is socially evaluated. Among such cues, gaze is particularly informative, as it serves as a window into another person's intentions, emotions, and evaluations.

Although prior research has examined whether social anxiety is associated with accuracy in judgments of others' mental states inferred from gaze, higher social anxiety has not been shown to improve overt performance in these tasks (Hezel and McNally 2014; Washburn et al. 2016). Attributing these performance outcomes to “over‐mentalizing” (Hezel and McNally 2014), this interpretation raises the possibility that downstream cognitive demands may obscure semantic processes underlying overt judgments, thereby attenuating evidence of enhanced mental state inference in measures of judgment accuracy.

To isolate the semantic processing involved in the inference of others' mental states from downstream mental operations, we focused on a neural index of semantic integration and assessed whether social anxiety predicts enhanced neural sensitivity to the semantic meaning conveyed by gaze. Specifically, we used the N400 as a neural index of semantic violation detection when a mental‐state word was presented following a gaze pattern. Given the prevailing view of social anxiety as disruptive, one might expect higher social anxiety to impair semantic integration of mental‐state meanings from gaze cues. However, this expectation may be premature if social anxiety facilitates the assessment of socially meaningful cues, a possibility that has received little empirical attention.

### 1.1 Social Anxiety and Mental State Inference

Although evidence for enhanced mental‐state inference in social anxiety remains limited, the possibility that social anxiety may facilitate semantic processing of socially meaningful information is consistent with evidence from a neuroscience study. McTeague et al. (2011) demonstrated that social anxiety is associated with sustained attention to socially relevant cues. Using steady‐state visual evoked potentials (ssVEPs) recorded through high‐density electroencephalogram (EEG), the researchers investigated how social anxiety modulates the perception of affective facial cues. Participants with high and low levels of social anxiety, assessed using the Liebowitz Social Anxiety Scale (Fresco et al. 2001)—a widely used measure of social anxiety—were shown flickering images of faces displaying neutral, happy, fearful, and angry expressions. The researchers found that individuals with high social anxiety exhibited heightened and sustained neural responses to emotional faces (both positive and negative) compared to neutral ones, while those with low social anxiety showed no such effect. Notably, this amplified neural response was specific to the occipital visual cortex, with a quick onset, persisting throughout the 3500‐millisecond viewing period. These findings suggest a broad and temporally sustained hyper‐attentiveness to facial affective cues associated with social anxiety.

McTeague et al.'s (2011) evidence suggests that social anxiety is linked to an enhancement, rather than an impairment, of visual processing that may support recognition of different emotional facial expressions. Here, we extended the prior work by examining whether such enhancement also supports the inference of others' mental states. Accordingly, we hypothesized that individuals higher in social anxiety may exhibit enhanced electrocortical sensitivity during the semantic processing of mental‐state meanings conveyed by gaze.

### 1.2 Present Investigation

To investigate whether social anxiety predicts enhanced neural sensitivity during the semantic integration of mental‐state meanings from gaze, we adopted the Reading the Mind in the Eyes (RME) test (Baron‐Cohen et al. 2001) and modified it into an electroencephalogram (EEG) paradigm designed to capture neural responses to semantic expectancy violations. In the conventional RME test, participants are shown a photographed gaze along with four mental state words—of which only one corresponds to the culturally consensual meaning of the mental state shown by the target gaze. Participants select one word (out of the four presented) that correctly describes the target's gaze expression. In the EEG‐adapted version, each gaze was presented one at a time and immediately followed by a single superimposed mental‐state adjective that was either consistent or inconsistent with the culturally shared meaning of the gaze. Participants' EEG was recorded, and we focused on an event‐related potential (ERP) component known as N400, to assess semantic incongruities associated with the gaze.

The N400 is a negative‐going component of the ERP that indexes semantic expectancy violations (Kutas and Hillyard 1980). It has been observed in linguistic contexts when an unexpected word is encountered (Kutas and Hillyard 1980), and in other experimental modalities involving pictures (Goto et al. 2009), mental state inference (Na and Kitayama 2011), and social norm violation (Salvador, Mu, et al. 2020). Here, the N400 reflects the degree of semantic incongruity between the adjective and the inferred mental state conveyed by the gaze. We predicted that incongruent words would elicit greater N400 responses than congruent words.

This paradigm allows us to examine whether social anxiety is associated with differences in neural sensitivity to semantic expectancy violations that arise during the processing of mental‐state meanings from gaze. One possibility is that higher social anxiety attenuates this neural sensitivity, in which case the N400 incongruity effect would decrease as social anxiety increases. An alternative possibility is that social anxiety enhances this neural sensitivity, in which case the N400 incongruity effect would increase with social anxiety. Importantly, these alternatives concern neural sensitivity during semantic integration, as indexed by the N400 incongruity effect, rather than differences in judgment accuracy or overall task performance.

Lastly, our hypothesis implies that, irrespective of its magnitude or direction, the effect of social anxiety on N400 responses should be specific to mental state inference—rather than reflecting a generalized sensitivity to semantic expectancy violations. This distinction is important as recent work suggests that anxiety can modulate N400 responses to semantic incongruity, even in nonsocial semantic contexts (Lepock et al. 2024). To determine whether the present effects of social anxiety are specific to the semantic processing of others' mental states, we included a control condition involving animal stereotypes that did not require mental state inference (Harkness et al. 2005). This control allowed us to disentangle N400 responses specific to mental state inference from those reflecting more general semantic associations. Lastly, we explored if we might detect similar effects in other late ERP components, especially late‐positive potential (LPP), which is linked to sustained engagement to an impinging stimulus often motivated by affective arousal (Cuthbert et al. 2000).

## 2 Method

### 2.1 Participants

The RME condition comprised a final sample of 57 participants (75% women, M_age_ = 18.79, SD_age_ = 0.88) who met the eligibility criteria for EEG procedure.^1^ An independent final sample of 50 participants (50% women, M_age_ = 18.82, SD_age_ = 1.00) who met the same eligibility criteria were recruited for the Control condition to serve as a comparison group.^2^ The sample size was based on recent work in social and cultural neuroscience, which finds reliable correlations between neural indices and theoretically‐relevant individual difference variables using 30 to 40 participants (Kraus and Kitayama 2019; Salvador, Kraus, et al. 2020; Salvador, Mu, et al. 2020). To ensure sufficient power, we sought at least 40 participants with analyzable data within each condition. All participants were right‐handed, had normal or corrected‐to‐normal vision, and had no current or history of medical conditions (head injury, concussion, seizure, epilepsy, psychiatric/neurological medications) that would render them unsuitable for the EEG procedure. Participants were screened for their ethnicity during recruitment: Only those with European heritage were recruited, as all gaze stimuli came from this cultural group (Baron‐Cohen et al. 2001). We limited our analysis to those who self‐identified as European Americans and had been born in the United States. This, in effect, ensured that all were native English speakers. Participants reported their gender as either male or female based on sex assigned at birth to be consistent with prior research in this area (Asher et al. 2017; Tsolaki et al. 2015; Washburn et al. 2016). This study was approved by the Institutional Review Board (IRB) at the University of Michigan. All participants provided informed consent prior to participation.

### 2.2 Procedure

The procedure in the RME condition was adapted from the original version (Baron‐Cohen et al. 2001) with several modifications. First, in the original task, each of a series of gaze cues is paired with four descriptive words, one of which correctly describes the mental state implied by the gaze. In the EEG‐adapted version, each gaze was paired with a single descriptive word and participants indicated whether or not the word was a correct description for the gaze. Second, in the original version, each gaze is paired with 3 incongruent words and 1 congruent word. Here, to maintain equal probabilities of congruent and incongruent words across trials, we replaced one incongruent word from each gaze stimulus with a new congruent word, which was synonymous to the original correct word, taken directly from the original task's glossary (Baron‐Cohen et al. 2001). A random number generator was used to determine which of the incongruent words to replace.

Similarly, the procedure in the Control condition was adapted from the original version of the validated control task, in which participants evaluate whether general trait adjectives correctly describe pictures of animals (Harkness et al. 2005). Specifically, we (1) replaced the original 12 images with higher‐resolution images, (2) replaced one incongruent word with a congruent word, using the same process described above, and (3) added 21 new images to parallel the total number of images in the RME condition. For each new image, we generated descriptive words that were pre‐tested for their accuracy in describing the animal (see Table S1 in Supporting Information).

The EEG paradigm was identical in both the RME and Control conditions. Participants saw each of the 33 images paired with each corresponding word (2 congruent, 2 incongruent) for a total of 132 trials. Image order was randomized, with all 33 images presented before any were repeated, and the order in which the 4 corresponding words were paired with each image was also randomized. Participants took self‐timed breaks after each set of 11 trials. Each trial began with a fixation cross, followed by the image. After 3 s, the image was superimposed by a descriptive word, which remained on the screen for 0.8 s. Following a 0.9‐s blank screen, participants were asked whether the word description was correct, and they responded ‘yes’ or ‘no’ on a keyboard using their left and right index fingers. Response keys were counterbalanced across participants. Figure 1A,B shows the task flow for RME and Control conditions, respectively.

**FIGURE 1:**
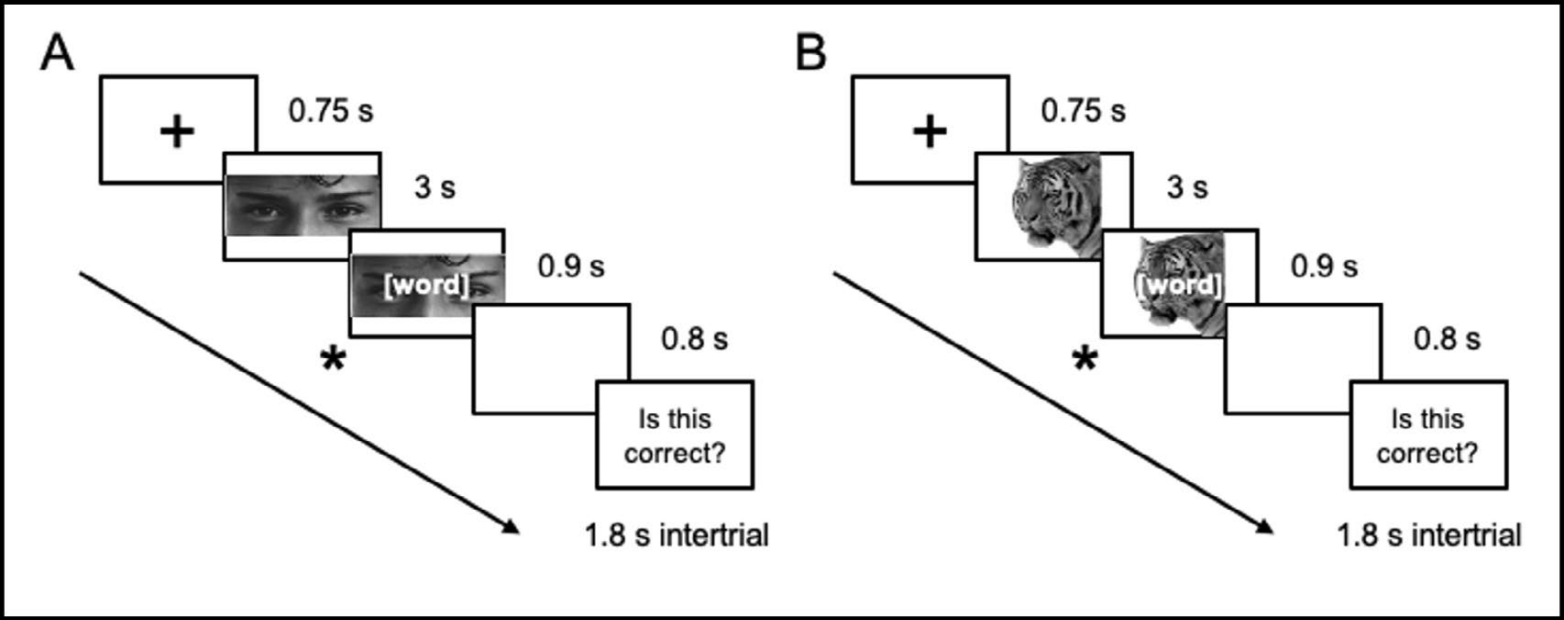
The rundown of trials in the (A) RME condition and (B) Control condition. The N400 was assessed time‐locked to the presentation of the word.

After the RME or Control task, participants completed the Liebowitz Social Anxiety Scale (LSAS‐SR) (Fresco et al. 2001), a measure of stable individual differences in social anxiety, in which they indicated how much fear or anxiety they generally feel across a broad range of social interaction and performance situations on a scale of 1 (none) to 4 (severe). In addition, participants completed several exploratory measures to rule out alternative explanations for any observed patterns (see Tables S4–S10 in Supporting Information), including measures of self‐construal (Singelis 1994) and five‐factor personality traits (Saucier 1994). Lastly, participants provided their demographic information, were thoroughly debriefed, and received either course credits or US $20 compensation for their participation.

### 2.3 EEG Recording and Preprocessing

EEG was recorded on a BioSemi Active Two System with 32 scalp channels configured to the 10–20 system.^3^ Four bipolar electrooculogram (EOG) electrodes were used to monitor vertical and horizontal eye movements. An electrode was attached to the left and right mastoids each to serve as offline reference. Impedances were kept within +/− 20 μV during the EEG setup.

Preprocessing was performed on MATLAB with EEGLAB plugin and ERPLAB extension. The EEG data were digitized at 512 Hz, resampled offline at 256 Hz, filtered offline (low‐pass: 20 Hz; high‐pass: 0.1 Hz), and re‐referenced to the average of the two mastoids. Poor EEG signals were interpolated spherically. Ocular artifacts were corrected using a regression‐based method (Gratton 1998). Next, ERPs were obtained by extracting 1000 ms epochs per correctly answered trial, time‐locked to the presentation of the descriptive word, which included a 200 ms pre‐stimulus period for baseline correction. Then, we conducted standard rejection procedures on the 32 scalp channels, in which trials were rejected for exceeding ±100 μV in a 200‐ms moving window with a 50‐ms step threshold, fluctuating more than 30 μV between two sampling points, or showing little to no activity (< 0.5 μV) throughout the trial (Luck 2014). These procedures resulted in the rejection of 2.75% congruent (RME: *M* = 3.72%, SD = 6.70%; Control: *M* = 1.64%, SD = 3.38%) and 2.83% incongruent (RME: *M* = 3.64%, SD = 7.13%; Control: *M* = 1.91%, SD = 3.55%) trials, respectively. Although none in the present study, participants with more than 50% trials rejected after the artifact rejection procedures would be excluded from analyses.

### 2.4 Data Analysis

Single‐trial ERPs were averaged across three midline centroparietal electrodes (Cz, CPz, and Pz), where N400 is typically maximal (Kutas and Federmeier 2011; see Figure 2). The maximum N400 peak, derived from the grand‐average waveform across all trials in the full sample, was visually identified and a 100‐ms time window was constructed around this peak to calculate the mean peak latency (435 ms).^4^ For each trial, mean amplitude was computed from a 100 ms window around the mean peak latency (i.e., from 385 to 485 ms). The N400 analyses included only correctly answered trials, as it is unclear what N400 indexes for incorrectly answered trials.

**FIGURE 2:**
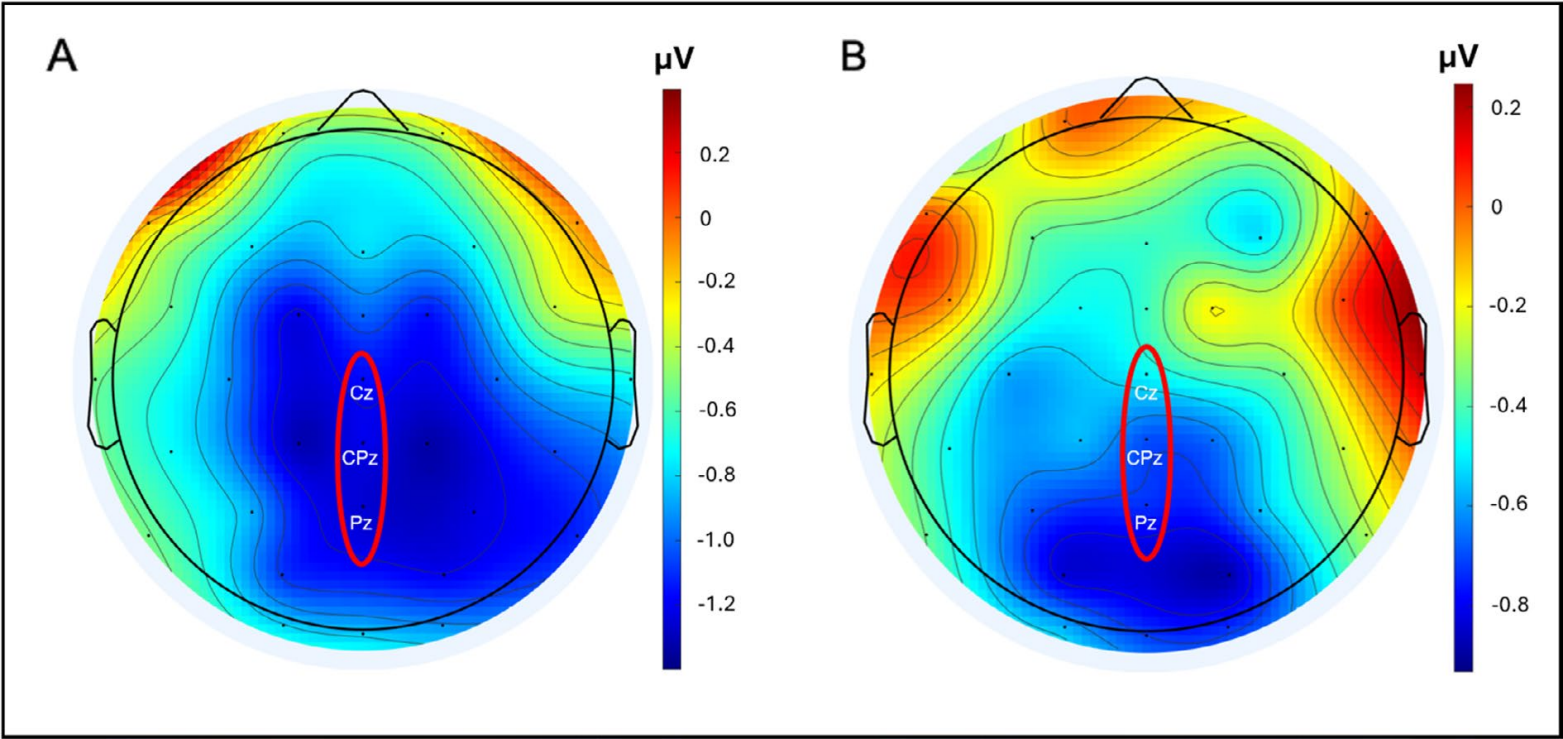
The scalp topography of the N400 mean amplitude difference between the incongruent and congruent trials in the (A) RME and (B) Control tasks. Scale is in μV and ranges from blue (negativity is greater for incongruent trials) to red (negativity is greater for congruent trials). Across both tasks, the hypothesized N400 effect (i.e., greater negativity for incongruent vs. congruent trials) is greatest at centroparietal sites, as in prior work (Kutas and Hillyard 1980). Thus, the N400 was analyzed by averaging the ERPs across Cz, CPz, and Pz.

We first conducted a set of analyses predicting accuracy in task performance from social anxiety and congruence, with sex as a covariate, for the RME and Control tasks separately. Next, we included both tasks in a single model to test the significance of the three‐way interaction. Finally, we conducted a parallel set of analyses predicting response time and N400. The analyses predicting accuracy included all non‐rejected ERP trials, whereas only correctly answered trials were included in the response time and N400 analyses.

All models included random intercepts for each subject and stimulus. Linear mixed‐effects models (LMMs) were fit to continuous outcomes (e.g., response time, N400) using restricted maximum likelihood estimation, whereas generalized linear mixed‐effects models (GLMMs) were used for binary outcomes (i.e., task accuracy) with a logit link function. All models were implemented in R using the lme4 package (Bates et al. 2015).

### 2.5 Exploratory Analysis

For exploratory purposes, we additionally performed the same set of analyses predicting Late Positive Potential (LPP) amplitude at the same midline centroparietal sites (Cz, CPz, and Pz), where the LPP tends to be maximal (Cuthbert et al. 2000; see Figure S2 in Supporting Information), within a 600–1000 ms time window after the word onset. To assess the robustness of the ERP findings, we further repeated the N400 and LPP analyses using a broader centroparietal cluster that included both midline and lateral electrodes.^5^ Across both N400 and LPP components, the results were consistent both when analyses were focused on midline centroparietal electrodes and when lateral centroparietal electrodes were included (see Tables S11 and S12, Figures S1–S3).

## 3 Results

### 3.1 Sample Characteristics

Participants in the RME and Control conditions reported similar levels of social anxiety (RME: *M* = 2.11, SD = 0.54; Control: *M* = 2.02, SD = 0.53), *t*(104) = −0.84, *p* = 0.40. There was no significant difference in age, *t*(98) = 0.17, *p* = 0.87, between the two conditions (RME: *M* = 18.79, SD = 0.88; Control: *M* = 18.82, SD = 1.00). However, there was a significant difference in sex proportion, χ^2^(1) = 6.38, *p* = 0.01. Preliminary analyses showed that sex had no effects on the dependent variable as either main effect or interactions (see Table S2 in Supporting Information), thus it was entered as a covariate in all analyses reported below. See Table 1 for descriptive statistics. The social anxiety scale had high reliability (RME: *α* = 0.81; Control: *α* = 0.80).

**TABLE 1:** Sample characteristics in the RME and Control conditions.

	RME	Control	Statistical comparison
Sample size	57	50	
Age	18.79 (0.88)	18.82 (1.00)	*t*(98) = 0.17, *p* = 0.87
Response time (milliseconds)	723 (341)	651 (303)	*t*(105) = −1.16, *p* = 0.25
Task performance	0.78 (0.05)	0.85 (0.06)	*t*(96) = 6.05, *p* < 0.001
Social anxiety	2.11 (0.54)	2.02 (0.53)	*t*(104) = −0.84, *p* = 0.40
Sex (male/female)	14/43	25/25	χ^2^(1) = 6.38, *p* = 0.01

*Note:* Values for Age, Response time, Task performance, and Social anxiety in the RME and Control columns represent sample means and standard deviations in parentheses.

### 3.2 Task Performance

Accuracy, as measured by the percentage of correctly answered trials, was high in both the RME and Control tasks, albeit higher in the latter (see Table 1). Social anxiety did not predict task performance in either task (RME: *b* = 0.07, 95% CI [−0.09, 0.23], *p* = 0.37; Control: *b* = −0.11, 95% CI [−0.39, 0.17], *p* = 0.42), and this relationship was similar across both tasks (Social Anxiety × Task: *b* = 0.19, 95% CI [−0.10, 0.48], *p* = 0.20).

### 3.3 Response Time

Response time, measured in milliseconds, was comparable in the RME and Control tasks (see Table 1). Social anxiety did not predict response time of correctly answered trials in either task (RME: *b* = −77.37, 95% CI [−210.67, 55.93], *p* = 0.27; Control: *b* = 17.65, 95% CI [−130.73, 166.02], *p* = 0.82), and this relationship was similar across both tasks (Social Anxiety × Task: *b* = −114.33, 95% CI [−308.25, 79.56], *p* = 0.26).

### 3.4 N400

N400 indexes the extent to which a target word was incongruent with a participant's mental state inference of the preceding gaze image or their judgment of the animal's general trait. Thus, a more negative N400 in the incongruent (vs. congruent) trials indicates greater neural sensitivity to semantic mismatch between the target word and the meaning inferred from the gaze or the animal's trait.

As expected, the N400 magnitude was more negative for incongruent relative to congruent trials in both RME and Control tasks, as shown in Figure 3A,B (RME: *b* = 1.26, 95% CI [0.53, 2.00], *p* < 0.001; Control: *b* = 1.20, 95% CI [0.48, 1.91], *p* = 0.001). The effect of congruence was not significantly different across the two tasks (Task × Congruence: *b* = 0.79, 95% CI [−0.94, 1.10], *p* = 0.88).

**FIGURE 3:**
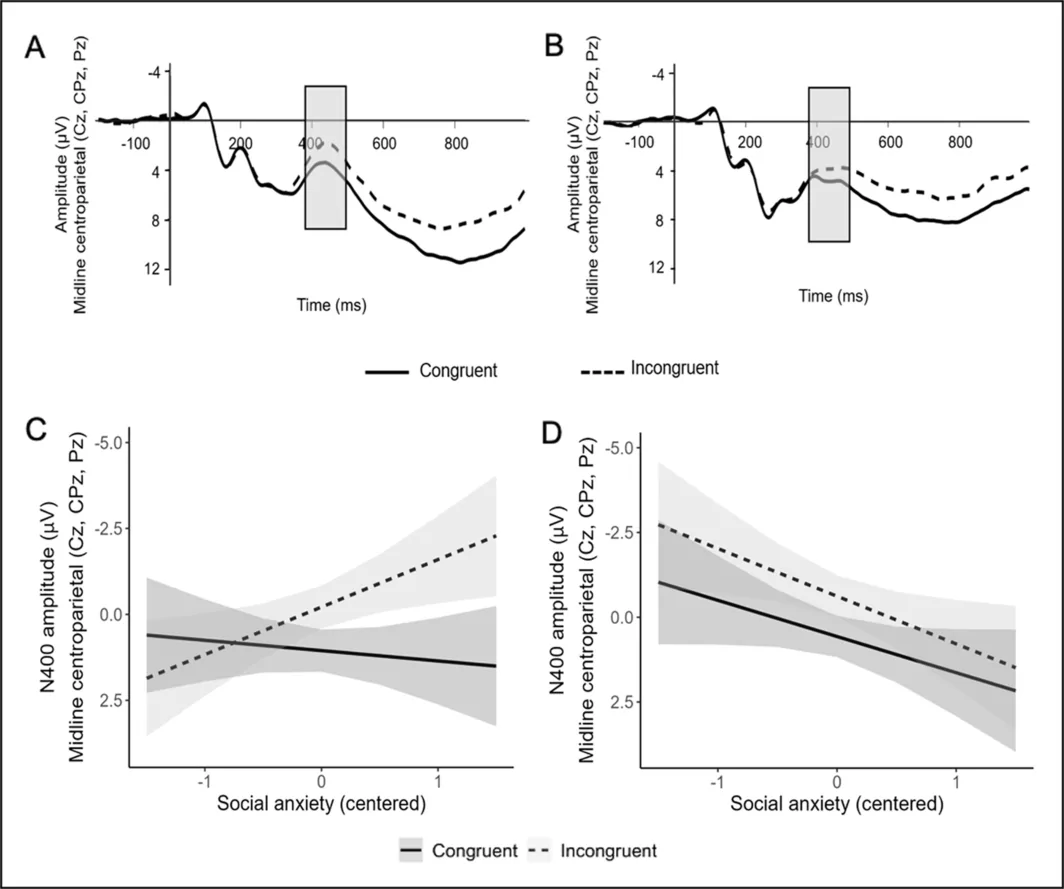
The grand averaged N400 waveforms from midline centroparietal electrodes (i.e., averaged from Cz, CPz, and Pz) in the (A) RME task and (B) Control task. The relationship between midline centroparietal N400 and social anxiety in the (C) RME task and (D) Control task. This relationship is only significant in the RME task. More negative N400 amplitudes indicate greater neural sensitivity to semantic incongruity. Error ribbons indicate standard error.

In the RME task, social anxiety moderated the magnitude of the N400 difference. As shown in Figure 3C, participants who reported higher levels of social anxiety showed a more negative N400 in response to the incongruent than congruent trials, *b* = 1.68, 95% CI [0.32, 3.03], *p* = 0.015. Furthermore, this pattern remained significant when controlling for a range of other individual difference measures and irrespective of gaze valence (see Tables S3–S10 in Supporting Information). In contrast, social anxiety was unrelated to the difference in N400 between congruent and incongruent trials in the Control task, as shown in Figure 3D, *b* = −0.34, 95% CI [−1.71, 1.02], *p* = 0.622. Thus, the relationship between social anxiety and N400 difference varied significantly across the two tasks (Social Anxiety × Congruence × Task: *b* = 2.02, 95% CI [0.09, 3.95], *p* = 0.040). This finding demonstrates that social anxiety predicted greater neural sensitivity in the semantic processing of social cue meanings that are specific to mental state (versus general trait) inference.

### 3.5 LPP

The LPP is a slow, positive‐going centroparietal ERP component that indexes attentive processing linked to affective arousal in response to emotionally salient stimuli (Cuthbert et al. 2000). In the current paradigm, we interpret LPP amplitude as indexing sustained affective and cognitive engagement to the expressive cues conveyed by the gaze or the semantic information conveyed by the animal image.

In both the RME and Control tasks, the LPP was greater for congruent relative to incongruent trials (RME: *b* = 2.48, 95% CI [1.65, 3.32], *p* < 0.001; Control: *b* = 1.81, 95% CI [0.95, 2.67], *p* < 0.001). This pattern, consistent across both tasks, suggests that successful semantic resolution, rather than incongruity per se, may drive later sustained processing in the present paradigm. Consistent with this, the effect of congruence did not vary significantly across the two tasks (Task × Congruence: *b* = 0.67, 95% CI [−0.54, 1.87], *p* = 0.283).

Yet, unlike the patterns observed with N400, social anxiety did not significantly modulate the LPP magnitudes in response to trial congruence in either the RME or Control task (RME: *b* = 1.36, 95% CI [−0.19, 2.90], *p* = 0.086; Control: *b* = 0.12, 95% CI [−1.54, 1.77], *p* = 0.890). Thus, the relationship between social anxiety and LPP magnitudes in congruent and incongruent trials did not vary significantly across the two tasks (Social Anxiety × Congruence × Task: *b* = 1.24, 95% CI [−1.02, 3.50], *p* = 0.283). This result may suggest that when the mental‐state adjective matched the meaning of the preceding stimulus, it elicited more sustained engagement independent of social anxiety (see Figure S3 in Supporting Information), although we note the possibility that LPP may, in part, reflect residual activity from the earlier N400 peak.

## 4 Discussion

### 4.1 Social Anxiety and Mental State Inference From Gaze

The central contribution of the present study is to demonstrate that social anxiety predicts enhanced neural sensitivity during the semantic integration of mental‐state meanings from gaze patterns. Specifically, social anxiety was associated with a larger N400 incongruity effect for gaze–word pairs, indicating greater neural sensitivity to semantic expectancy violations when the meaning inferred from a gaze conflicted with a subsequently presented mental‐state word. Importantly, this effect appeared specific to mental state inference: No corresponding modulation of the N400 by social anxiety was observed for semantic incongruity involving animal stereotypes. This specificity strengthens the interpretation that social anxiety is linked to differences in semantic processing of mental‐state meanings, rather than to general semantic processing tendencies.

Notably, social anxiety did not predict accuracy or response latency in explicit mental‐state judgments. This dissociation suggests that social anxiety‐related differences are evident in neural sensitivity during semantic integration, but not in the eventual judgment of mental‐state meanings from gaze. In other words, individuals high in social anxiety are no better at accurately judging the mental‐state meanings from gaze, despite an enhancement in neural sensitivity indexed by the N400. Prior research suggested that this absence of performance enhancement may reflect a pattern of “over‐mentalizing” (Hezel and McNally 2014) known to impair judgment (Wilson and Schooler 1991). It is plausible that such downstream cognitive processes may obscure enhanced semantic processing at the level of explicit judgment, yielding no corresponding advantage in performance.

An exploratory analysis performed on the LPP showed that this component is significantly greater for congruent (vs. incongruent) trials regardless of social anxiety. Moreover, this pattern was evident in both tasks. Given the robust N400 incongruity effect and the relatively early time window used to quantify the LPP (600–1000 ms), the LPP difference may, in part, reflect residual activity carried over from earlier semantic processing indexed by the N400. However, several features of the data suggest that the LPP effect may not be reducible to a simple continuation of the N400. Whereas the N400 was modulated by task demands and individual differences in social anxiety, the LPP showed neither task‐ nor anxiety‐related modulation. This dissociation raises the possibility that the LPP in this context reflects a distinct process, such as the affective or motivational significance of a successfully resolved semantic prediction—an avenue warranting future investigation.

The present findings are broadly consistent with prior neuroimaging research linking higher social anxiety to amplified and sustained neural engagement with affective facial expressions in visual cortex (McTeague et al. 2011), indicating heightened responsiveness to socially relevant information. Extending this work, the current study shows that social anxiety‐related differences are also observable at the level of semantic integration of mental‐state meanings from gaze. Accordingly, it is plausible that enhanced visual sensitivity may provide a clearer and more robust input to subsequent semantic processing. Future work is needed to establish this link.

### 4.2 Functional Benefits?

Given the scope of the present data, our conclusion is limited to the association between social anxiety and enhanced neural sensitivity during mental state inference. Thus, the functional significance of this sensitivity remains to be established. Nevertheless, such enhancement could plausibly facilitate vigilance to socially consequential information, including social evaluations, others' intentions, and potential social risks. In some circumstances, this vigilance could support avoidance of socially costly situations or adjustment to unfolding situational demands (Leary and Jongman‐Sereno 2014). In others, however, heightened vigilance may impose costs by increasing cognitive load and fostering counterproductive deliberation (Hezel and McNally 2014; Hofmann 2007; Rapee and Heimberg 1997). Thus, the functional consequences of social anxiety are likely to be context‐dependent.

In our recent study, Melani and Kitayama (2026) addressed this possibility by examining whether the longer‐term health implications of social anxiety vary as a function of societal norm strength. We found that social anxiety predicted lower biological health risk in Japan—where social norms are tightly enforced and deviations from normative expectations carry serious social consequences—but not in the United States, where norms are relatively loose and less strictly enforced. This finding provides initial support for the proposition that the consequences of social anxiety are likely to depend on the context in which it operates.

### 4.3 Limitations and Conclusions

Several important limitations of the present findings warrant emphasis. First, the present study was restricted to European American participants, as all gaze stimuli came from this group. Future research is needed to determine whether the observed effects would generalize to other cultural contexts. Second, although the Control task provided a comparison with semantic processing in a condition that did not require mental state inference, it may not fully isolate all processes that distinguish social from non‐social meaning. Future work must test a broader range of domains to better specify the boundary conditions. Third and relatedly, this effort may benefit from the use of different methodological parameters including a within‐subjects design. Fourth, future studies incorporating complementary measures of anxiety, such as the State–Trait Anxiety Inventory (STAI), may help distinguish the respective contributions of social anxiety and more generalized anxiety to semantic processing. Lastly, while we interpret the increased N400 amplitude as reflecting enhanced neural sensitivity to semantic incongruity, it is important to note that amplitude measures do not directly index processing speed. Future research using latency measures or time‐constrained behavioral paradigms could more directly assess the temporal dynamics of semantic integration in social anxiety.

Despite these limitations, our work used EEG markers and showed that social anxiety is associated with enhanced neural sensitivity to semantic integration during mental state inference linked to gaze patterns. Although the functional consequences of this enhanced sensitivity remain to be established, identifying it provides an important basis for investigating the possibility that a psychological disposition known primarily for its costs may also confer context‐dependent advantages in processing socially consequential information.

## Author Contributions

**Irene Melani:** conceptualization, methodology, data curation, investigation, validation, formal analysis, visualization, project administration, writing – original draft, writing – review and editing. **Martha K. Berg:** conceptualization, methodology, data curation, investigation, validation, formal analysis, visualization, project administration, writing – original draft, writing – review and editing. **James Glazer:** formal analysis, validation, supervision, writing – review and editing. **Shinobu Kitayama:** conceptualization, methodology, supervision, funding acquisition, writing – original draft, writing – review and editing, resources.

## Funding

This work was supported by grants from the National Science Foundation (BCS 0717982 and SES 1325881) awarded to Shinobu Kitayama.

## Conflicts of Interest

The authors declare no conflicts of interest.

## Supporting information


**Figure S1:** The grand averaged N400 waveforms from midline and lateral centroparietal electrodes (i.e., averaged from Cz, C3, C4, CPz, CP1, CP2, CP5, CP6, Pz, P3, P4, P7, and P8) in the (A) RME task and (B) Control task. The relationship between midline and lateral centroparietal N400 and social anxiety in the (C) RME task and (D) Control task. This relationship is only significant in the RME task. Error ribbons indicate standard error.
**Figure S2:** The scalp topography of the LPP mean amplitude. Panels (A) and (B) show the LPP for congruent and incongruent trials in the RME task, respectively. Panels (C) and (D) show the LPP for congruent and incongruent trials in the Control task, respectively. Scale is in μV and ranges from red (higher positivity) to blue (lower positivity). As in prior work (Cuthbert et al. 2000), the LPP is pronounced across the centroparietal sites. The LPP was analyzed by averaging the ERPs at the midline electrodes (Cz, CPz, and Pz) and across an extended centroparietal cluster including lateral sites.
**Figure S3:** The relationship between social anxiety and centroparietal LPP. Panels (A) and (B) show midline LPP in the RME and Control tasks, respectively. Panels (C) and (D) show midline and lateral LPP in the RME and Control tasks, respectively. The relationship is marginal in the RME task. The social anxiety × congruence × task three‐way interaction did not reach statistical significance. Error ribbons indicate standard error.
**Table S1:** Control task stimuli.
**Table S2:** Exploratory analysis of sex as a moderator of the relationship between social anxiety and improved mental state processing.
**Table S3:** Exploratory analysis of valence as a moderator of the relationship between social anxiety and improved mental state processing.
**Table S4:** Exploratory analysis controlling for Independent Self‐Construal^a^.
**Table S5:** Exploratory analysis controlling for Interdependent Self‐Construal^a^.
**Table S6:** Exploratory analysis controlling for Openness to Experience subscale of the Big‐Five personality trait^a^.
**Table S7:** Exploratory analysis controlling for Conscientiousness subscale of the Big‐Five personality trait^a^.
**Table S8:** Exploratory analysis controlling for Extraversion subscale of the Big‐Five personality trait^a^.
**Table S9:** Exploratory analysis controlling for Agreeableness subscale of the Big‐Five personality trait^a^.
**Table S10:** Exploratory analysis controlling for Neuroticism subscale of the Big‐Five personality trait^a^.
**Table S11:** Exploratory analysis predicting N400 of correctly answered trials at midline and lateral centroparietal sites as a function of social anxiety, congruence, and task.
**Table S12:** Exploratory analysis predicting Late Positive Potential (600–1000 ms) of correctly answered trials at midline and lateral centroparietal sites as a function of social anxiety, congruence, and task.
**Table S13:** Exploratory analysis predicting N400 of correctly answered trials at midline centroparietal sites using adjusted peak‐latency time windows as a function of social anxiety, congruence, and task.
**Table S14:** Exploratory analysis predicting N400 of correctly answered trials at midline and lateral centroparietal sites using adjusted peak‐latency time windows as a function of social anxiety, congruence, and task.

## Data Availability

Deidentified data and R codes are available on Open Science Framework (https://osf.io/tw5rp/).

## Endnotes

^1^
6 participants who did not meet the eligibility criteria were excluded from the study (2 for history of concussion, 1 for history of seizure, 2 of non‐European descent [so as to minimize cultural variation in the inference of mental state meanings associated with gaze expressions], 1 for current psychiatric medication) and an additional 5 participants were excluded from the analyses (2 for technical issues with the EEG equipment and 3 for missing social anxiety data).

^2^
Eight participants who did not meet the eligibility criteria were excluded from the study (5 for history of concussion, 1 for history of seizure, 2 of non‐European descent) and an additional 2 participants were excluded from analyses due to EEG malfunction.

^3^
The 32 scalp channels were Fp1, Fp2, F7, F3, Fz, F4, F8, FC5, FC1, FCz, FC2, FC6, C3, Cz, C4, T7, T8, CP5, CP1, CPz, CP2, CP6, P7, P3, Pz, P4, P8, PO3, PO4, O1, Oz, and O2.

^4^
A parallel set of analyses was performed in which, for the RME and Control tasks, N400 amplitudes were extracted based on the corresponding peak latency windows identified separately for participants high and low in social anxiety. See Tables S13 and S14 in Supporting Information for results of the analyses.

^5^
Midline and lateral centroparietal electrodes included Cz, C3, C4, CPz, CP1, CP2, CP5, CP6, Pz, P3, P4, P7, and P8.
