# Supplementary material for: Social Anxiety Predicts Enhanced Electrocortical Sensitivity to the Mental State Meanings Revealed by Gaze Patterns
Source: Psychophysiology. 2026 Sep 28;63(10):e70409. doi: 10.1111/psyp.70409 (PMC13620438; doi:10.1111/psyp.70409)
Supplement: Supplementary file 1 [file PSYP-63-e70409-s001.docx]

**Social Anxiety Predicts Enhanced Electrocortical Sensitivity to the Mental State Meanings Revealed by Gaze Patterns**

Irene Melani, Martha K. Berg, James Glazer, and Shinobu Kitayama

**Supplementary Materials**

**Exploratory Analyses**

**Sex Effects**

The interaction between social anxiety and congruence to predict N400 in the RME task was consistent whether or not sex was included as a covariate. Furthermore, an exploratory analysis revealed that sex did not significantly interact with any other predictors in the model (see Table S2).

**Valence Effects**

The interaction between social anxiety and congruence to predict N400 in the RME task remained significant, regardless of valence. Valence did not significantly interact with social anxiety and congruence to predict differences in N400 (see Table S3).

**Individual Difference Measures**

We tested whether any of the individual differences we assessed (2 self-construal dimensions and 5 personality dimensions) could explain the relationship between social anxiety and increased N400 for incongruent trials in the RME task. For each measure, we estimated a model that controlled for the interaction between the individual difference measure and congruence. None of the tested measures predicted N400, nor did any interact significantly with congruence to predict N400. The social anxiety × congruence interaction remained consistent across all models (see Tables S4 to S10).

**N400 and LPP**

We conducted parallel analyses predicting N400 and Late Positive Potential (LPP) amplitudes on correctly answered trials as a function of social anxiety, congruence, task, and their interactions at centroparietal sites. Analyses were performed at: (1) midline centroparietal electrodes (Cz, CPz, Pz) and (2) an expanded set of midline and lateral centroparietal electrodes (Cz, C3, C4, CPz, CP1, JCP2, CP5, CP6, Pz, P3, P4, P7, P8).

For N400, results were consistent across both sets of electrode sites. Specifically, social anxiety predicted larger N400 amplitudes for incongruent (vs. congruent) trials in the RME condition, whereas no such effect emerged in the Control condition, both when analyses were restricted to midline centroparietal sites (see Results in main article) and when lateral centroparietal sites were included (see Table S11 and Figure S1).

For LPP, the three-way interaction among social anxiety, congruence, and task was not significant at either the midline or the expanded centroparietal sites that included lateral electrodes, indicating that social anxiety did not reliably modulate LPP amplitudes across tasks (see Table S12 and Figures S2 to S3).

**N400 Peak Latency**

To address potential differences in N400 peak latency across levels of social anxiety, we repeated the primary analyses using N400 peak latency windows identified separately for participants high and low in social anxiety based on a median split. The results remained consistent: Social anxiety predicted a more negative N400 incongruity effect in the RME task but not in the Control task (see Tables S13 and S14).

**Table S1.**

*Control task stimuli.*

New stimuli (P2, P3, and 12-33) were normed with 52 participants on Amazon Mechanical Turk, who responded on a scale from 1 *(Not well at all)* to 6 *(Extremely well)* to the question: *How well do the following words describe this animal?* Bolded words are those deemed “correct”. Words that were rated differently than they were intended (e.g., a “correct” description rated below the midpoint of the scale) were replaced and retested with a new sample of 51 participants. Final words and ratings are listed below.

| Stimulus Number | Animal | Description | Mean Rating (SD) |
| --- | --- | --- | --- |
| P1 | Cat (practice trial) | **Independent** |  |
|  |  | Clumsy |  |
| P2 | Donkey (practice trial) | **Stubborn** |  |
|  |  | Cunning |  |
| P3 | Chicken (practice trial) | **Skittish** |  |
|  |  | Brave |  |
| 1 | Monkey | **Playful** |  |
|  |  | **Fun** |  |
|  |  | Tame |  |
|  |  | Alert |  |
| 2 | Tiger | **Fierce** |  |
|  |  | **Ferocious** |  |
|  |  | Timid |  |
|  |  | Obedient |  |
| 3 | Deer | **Timid** |  |
|  |  | **Shy** |  |
|  |  | Temperamental |  |
|  |  | Clever |  |
| 4 | Lion | **Brave** |  |
|  |  | **Fearless** |  |
|  |  | Gentle |  |
|  |  | Patient |  |
| 5 | Cheetah | **Agile** |  |
|  |  | **Quick** |  |
|  |  | Quiet |  |
|  |  | Frail |  |
| 6 | Dog | **Friendly** |  |
|  |  | **Sociable** |  |
|  |  | Graceful |  |
|  |  | Savage |  |
| 7 | Raccoon | **Mischievous** |  |
|  |  | **Troublemaking** |  |
|  |  | Agile |  |
|  |  | Stubborn |  |
| 8 | Elephant | **Lethargic** |  |
|  |  | **Sluggish** |  |
|  |  | Ferocious |  |
|  |  | Attentive |  |
| 9 | Owl | **Alert** |  |
|  |  | **Watchful** |  |
|  |  | Bold |  |
|  |  | Restless |  |
| 10 | Snake | **Sly** |  |
|  |  | **Stealthy** |  |
|  |  | Playful |  |
|  |  | Bashful |  |
| 11 | Dolphin | **Graceful** |  |
|  |  | **Acrobatic** |  |
|  |  | Hostile |  |
|  |  | Sly |  |
| 12 | Butterfly | **Delicate** | **5.13 (1.07)** |
|  |  | **Fragile** | **5.08 (1.13)** |
|  |  | Clever | 2.75 (1.77) |
|  |  | Stubborn | 2.06 (1.59) |
| 13 | Beaver | **Hardworking** | **4.60 (1.46)** |
|  |  | **Diligent** | **4.23 (1.54)** |
|  |  | Savage | 2.35 (1.50) |
|  |  | Lazy |  |
| 14 | Bear | **Rugged** |  |
|  |  | **Tough** |  |
|  |  | Delicate |  |
|  |  | Cowardly |  |
| 15 | Giraffe | **Gentle** | **4.13 (1.58)** |
|  |  | **Calm** | **4.40 (1.49)** |
|  |  | Hostile | 2.22 (1.50) |
|  |  | Courageous | 2.75 (1.52) |
| 16 | Cow | **Mellow** | **4.25 (1.45)** |
|  |  | **Calm** |  |
|  |  | Quick | 2.42 (1.56) |
|  |  | Ferocious | 2.25 (1.63) |
| 17 | Dove | **Peaceful** | **4.33 (1.63)** |
|  |  | **Serene** | **4.42 (1.53)** |
|  |  | Stealthy | 2.42 (1.58) |
|  |  | Aggressive | 2.25 (1.61) |
| 18 | Fox | **Cunning** | **4.31 (1.48)** |
|  |  | **Sly** | **4.27 (1.43)** |
|  |  | Obedient | 2.19 (1.56) |
|  |  | Friendly | 2.69 (1.75) |
| 19 | Sheep | **Passive** |  |
|  |  | **Meek** |  |
|  |  | Disagreeable |  |
|  |  | Majestic |  |
| 20 | Gorilla | **Strong** |  |
|  |  | **Powerful** |  |
|  |  | Aloof |  |
|  |  | Shy |  |
| 21 | Horse | **Noble** | **4.46 (1.38)** |
|  |  | **Dignified** | **4.40 (1.36)** |
|  |  | Mischievous | 2.69 (1.62) |
|  |  | Frail | 2.31 (1.63) |
| 22 | Eagle | **Majestic** |  |
|  |  | **Regal** |  |
|  |  | Lazy |  |
|  |  | Playful |  |
| 23 | Mouse | **Quiet** | **4.15 (1.32)** |
|  |  | **Silent** | **3.87 (1.57)** |
|  |  | Sluggish | 2.12 (1.60) |
|  |  | Fierce | 2.27 (1.47) |
| 24 | Otter | **Lively** |  |
|  |  | **Playful** |  |
|  |  | Peaceful |  |
|  |  | Proud |  |
| 25 | Parrot | **Talkative** |  |
|  |  | **Loud** |  |
|  |  | Shy |  |
|  |  | Strong |  |
| 26 | Peacock | **Proud** |  |
|  |  | **Boastful** |  |
|  |  | Rugged |  |
|  |  | Hardworking |  |
| 27 | Pig | **Greedy** |  |
|  |  | **Gluttonous** |  |
|  |  | Elegant |  |
|  |  | Agile |  |
| 28 | Rabbit | **Quick** |  |
|  |  | **Nimble** |  |
|  |  | Patient |  |
|  |  | Arrogant |  |
| 29 | Skunk | **Defensive** |  |
|  |  | **Protective** |  |
|  |  | Greedy |  |
|  |  | Friendly |  |
| 30 | Shark | **Dangerous** |  |
|  |  | **Deadly** |  |
|  |  | Timid |  |
|  |  | Passive |  |
| 31 | Sloth | **Lazy** |  |
|  |  | **Inactive** |  |
|  |  | Diligent |  |
|  |  | Aggressive |  |
| 32 | Swan | **Elegant** |  |
|  |  | **Refined** |  |
|  |  | Restless |  |
|  |  | Courageous |  |
| 33 | Turtle | **Slow** |  |
|  |  | **Unhurried** |  |
|  |  | Temperamental |  |
|  |  | Mischievous |  |

**Table S2.**

*Exploratory analysis of sex as a moderator of the relationship between social anxiety and improved mental state processing.*

| Effect | Estimate | *SE* | 95% CI | | *p* |
| --- | --- | --- | --- | --- | --- |
|  |  |  | *LL* | *UL* |  |
| Fixed effects |  |  |  |  |  |
| Intercept | 0.27 | 0.76 | -1.19 | 1.74 | .721 |
| Sex ^a^ | -1.51 | 1.49 | -4.37 | 1.35 | .316 |
| Social anxiety ^b^ | -1.25 | 1.41 | -3.95 | 1.46 | .381 |
| Congruence ^c^ | 1.67 | 0.49 | 0.72 | 2.62 | < .001 |
| Sex × social anxiety | 2.79 | 2.82 | -2.63 | 8.21 | .326 |
| Sex × congruence | -1.32 | 0.97 | -3.23 | 0.57 | .171 |
| Social anxiety × congruence | 2.27 | 0.91 | 0.48 | 4.05 | .013 |
| Sex × social anxiety × congruence | -1.08 | 1.83 | -4.66 | 2.50 | .552 |
| Random effects |  |  |  |  |  |
| Within-subject variance | 16.46 |  | 10.17 | 23.42 |  |
| Within-stimulus variance | 0.96 |  | 0.20 | 2.41 |  |

*Note.* Number of subjects = 57, number of stimuli = 33, total *N* = 5,674. CI = confidence interval; *LL* = lower limit; *UL* = upper limit.

^a^ -0.5 = male, 0.5 = female. ^b^ Social anxiety was centered within each task. ^c^ -0.5 = incongruent, 0.5 = congruent.

**Table S3.**

*Exploratory analysis of valence as a moderator of the relationship between social anxiety and improved mental state processing.*

| Effect | Estimate | *SE* | 95% CI | | *p* |
| --- | --- | --- | --- | --- | --- |
|  |  |  | *LL* | *UL* |  |
| Fixed effects |  |  |  |  |  |
| Intercept | 0.86 | 0.72 | -0.52 | 2.25 | .233 |
| Sex *^a^* | -2.08 | 1.38 | -4.76 | 0.59 | .137 |
| Social anxiety ^b^ | 0.13 | 1.14 | -2.07 | 2.34 | .908 |
| Valence (0) ^c^ | -0.77 | 1.09 | -2.90 | 1.36 | .487 |
| Valence (1) ^c^ | -0.70 | 0.56 | -1.78 | 0.39 | .222 |
| Congruence ^d^ | 0.60 | 0.47 | -0.32 | 1.53 | .201 |
| Social anxiety × congruence | 1.95 | 0.87 | 0.25 | 3.65 | .025 |
| Social anxiety × valence (0) | -1.72 | 1.50 | -4.66 | 1.21 | .252 |
| Social anxiety × valence (1) | -1.33 | 0.75 | -2.81 | 0.15 | .078 |
| Valence (0) × congruence | 1.35 | 1.62 | -1.82 | 4.51 | .405 |
| Valence (1) × congruence | 1.88 | 0.81 | 0.29 | 3.47 | .021 |
| Valence (0) × social anxiety × congruence | 4.44 | 3.00 | -1.43 | 10.31 | .139 |
| Valence (1) × social anxiety × congruence | -1.69 | 1.51 | -4.64 | 1.27 | .262 |
| Random effects |  |  |  |  |  |
| Within-subject variance | 16.47 |  | 10.40 | 23.92 |  |
| Within-stimulus variance | 0.99 |  | 0.14 | 2.23 |  |

*Note.* Number of subjects = 57, number of stimuli = 33, total *N* = 5,674. CI = confidence interval; *LL* = lower limit; *UL* = upper limit.

*^a^* -0.5 = male, 0.5 = female. ^b^ Social anxiety was centered within each task. ^c^ Valence was determined based on prior research using the same stimuli (Hezel & McNally, 2014); -1 (reference) = negative, 0 = neutral, 1 = positive. ^d^ 0.5 = incongruent, 0.5 = congruent.

**Table S4.**

*Exploratory analysis controlling for Independent Self-Construal ^a^*

| Effect | Estimate | *SE* | 95% CI | | *p* |
| --- | --- | --- | --- | --- | --- |
|  |  |  | *LL* | *UL* |  |
| Fixed effects |  |  |  |  |  |
| Intercept | 0.66 | 0.70 | -0.69 | 2.01 | .349 |
| Sex ^b^ | -2.05 | 1.41 | -4.75 | 0.66 | .152 |
| Social anxiety ^c^ | -0.26 | 1.29 | -2.73 | 2.22 | .842 |
| Independent self-construal ^d^ | 0.03 | 0.92 | -1.73 | 1.79 | .974 |
| Congruence ^e^ | 1.31 | 0.38 | 0.57 | 2.05 | < .001 |
| Social anxiety × congruence | 1.74 | 0.79 | 0.19 | 3.28 | .028 |
| Independent self-construal × congruence | -0.04 | 0.59 | -1.19 | 1.12 | .947 |
| Random effects |  |  |  |  |  |
| Within-subject variance | 16.86 |  | 10.37 | 24.03 |  |
| Within-stimulus variance | 1.03 |  | 0.23 | 2.54 |  |

*Note.* Number of subjects = 56, number of stimuli = 33, total *N* = 5,582. CI = confidence interval; *LL* = lower limit; *UL* = upper limit.

*^a^* (Singelis, 1994) ^b^ -0.5 = male, 0.5 = female. ^c^ Social anxiety was centered within each task. ^d^ Independent self-construal was centered within each task. ^e^ 0.5 = incongruent, 0.5 = congruent.

**Table S5.**

*Exploratory analysis controlling for Interdependent Self-Construal ^a^*

| Effect | Estimate | *SE* | 95% CI | | *p* |
| --- | --- | --- | --- | --- | --- |
|  |  |  | *LL* | *UL* |  |
| Fixed effects |  |  |  |  |  |
| Intercept | 0.54 | 0.69 | -0.79 | 1.87 | .439 |
| Sex ^b^ | -2.19 | 1.38 | -4.85 | 0.47 | .119 |
| Social anxiety ^c^ | -0.45 | 1.11 | -2.59 | 1.69 | .685 |
| Interdependent self-construal ^d^ | 0.36 | 0.88 | -1.34 | 2.05 | .686 |
| Congruence ^e^ | 1.20 | 0.38 | 0.47 | 1.94 | < .001 |
| Social anxiety × congruence | 1.59 | 0.69 | 0.23 | 2.94 | .022 |
| Independent self-construal × congruence | 0.50 | 0.58 | -0.62 | 1.63 | .381 |
| Random effects |  |  |  |  |  |
| Within-subject variance | 16.60 |  | 10.21 | 23.67 |  |
| Within-stimulus variance | 0.90 |  | 0.16 | 2.30 |  |

*Note.* Number of subjects = 56, number of stimuli = 33, total *N* = 5,572. CI = confidence interval; *LL* = lower limit; *UL* = upper limit.

*^a^* (Singelis, 1994) ^b^ -0.5 = male, 0.5 = female. ^c^ Social anxiety was centered within each task. ^d^ Interdependent self-construal was centered within each task. ^e^ 0.5 = incongruent, 0.5 = congruent.

**Table S6.**

*Exploratory analysis controlling for Openness to Experience subscale of the Big-Five personality trait ^a^*

| Effect | Estimate | *SE* | 95% CI | | *p* |
| --- | --- | --- | --- | --- | --- |
|  |  |  | *LL* | *UL* |  |
| Fixed effects |  |  |  |  |  |
| Intercept | 0.61 | 0.70 | -0.73 | 1.94 | .386 |
| Sex ^b^ | -2.09 | 1.39 | -4.77 | 0.59 | .140 |
| Social anxiety ^c^ | -0.40 | 1.16 | -2.64 | 1.83 | .729 |
| Openness to experience ^d^ | -0.07 | 0.67 | -1.36 | 1.23 | .923 |
| Congruence ^e^ | 1.26 | 0.37 | 0.53 | 1.99 | < .001 |
| Social anxiety × congruence | 1.73 | 0.72 | 0.32 | 3.14 | .016 |
| Openness to experience × congruence | 0.11 | 0.43 | -0.74 | 0.96 | .800 |
| Random effects |  |  |  |  |  |
| Within-subject variance | 16.78 |  | 10.37 | 23.86 |  |
| Within-stimulus variance | 0.97 |  | 0.20 | 2.42 |  |

*Note.* Number of subjects = 57, number of stimuli = 33, total *N* = 5,674. CI = confidence interval; *LL* = lower limit; *UL* = upper limit.

*^a^* (Saucier, 1994) ^b^ -0.5 = male, 0.5 = female. ^c^ Social anxiety was centered within each task. ^d^ Openness to Experience was centered within each task. ^e^ 0.5 = incongruent, 0.5 = congruent.

**Table S7.**

*Exploratory analysis controlling for Conscientiousness subscale of the Big-Five personality trait ^a^*

| Effect | Estimate | *SE* | 95% CI | | *p* |
| --- | --- | --- | --- | --- | --- |
|  |  |  | *LL* | *UL* |  |
| Fixed effects |  |  |  |  |  |
| Intercept | 0.75 | 0.70 | -0.61 | 2.10 | .292 |
| Sex ^b^ | -2.65 | 1.49 | -5.51 | 0.21 | .081 |
| Social anxiety ^c^ | -0.02 | 1.16 | -2.26 | 2.21 | .983 |
| Conscientiousness ^d^ | 0.58 | 0.60 | -0.56 | 1.73 | .332 |
| Congruence ^e^ | 1.26 | 0.37 | 0.53 | 2.00 | < .001 |
| Social anxiety × congruence | 1.64 | 0.70 | 0.26 | 3.01 | .020 |
| Conscientiousness × congruence | -0.14 | 0.36 | -0.84 | 0.57 | .703 |
| Random effects |  |  |  |  |  |
| Within-subject variance | 16.47 |  | 10.17 | 23.43 |  |
| Within-stimulus variance | 0.97 |  | 0.20 | 2.42 |  |

*Note.* Number of subjects = 57, number of stimuli = 33, total *N* = 5,674. CI = confidence interval; *LL* = lower limit; *UL* = upper limit.

*^a^* (Saucier, 1994) ^b^ -0.5 = male, 0.5 = female. ^c^ Social anxiety was centered within each task. ^d^ Conscientiousness was centered within each task. ^e^ 0.5 = incongruent, 0.5 = congruent.

**Table S8.**

*Exploratory analysis controlling for Extraversion subscale of the Big-Five personality trait ^a^*

| Effect | Estimate | *SE* | 95% CI | | *p* |
| --- | --- | --- | --- | --- | --- |
|  |  |  | *LL* | *UL* |  |
| Fixed effects |  |  |  |  |  |
| Intercept | 0.66 | 0.70 | -0.68 | 2.00 | .349 |
| Sex ^b^ | -2.30 | 1.41 | -5.02 | 0.41 | .110 |
| Social anxiety ^c^ | 0.13 | 1.30 | -2.38 | 2.63 | .923 |
| Extraversion ^d^ | 0.33 | 0.47 | -0.58 | 1.23 | .492 |
| Congruence ^e^ | 1.27 | 0.37 | 0.54 | 2.00 | < .001 |
| Social anxiety × congruence | 2.28 | 0.79 | 0.72 | 3.83 | .004 |
| Extraversion × congruence | 0.45 | 0.30 | -0.14 | 1.04 | .132 |
| Random effects |  |  |  |  |  |
| Within-subject variance | 16.58 |  | 10.24 | 23.58 |  |
| Within-stimulus variance | 0.98 |  | 0.21 | 2.43 |  |

*Note.* Number of subjects = 57, number of stimuli = 33, total *N* = 5,674. CI = confidence interval; *LL* = lower limit; *UL* = upper limit.

*^a^* (Saucier, 1994) ^b^ -0.5 = male, 0.5 = female. ^c^ Social anxiety was centered within each task. ^d^ Extraversion was centered within each task. ^e^ 0.5 = incongruent, 0.5 = congruent.

**Table S9.**

*Exploratory analysis controlling for Agreeableness subscale of the Big-Five personality trait ^a^*

| Effect | Estimate | *SE* | 95% CI | | *p* |
| --- | --- | --- | --- | --- | --- |
|  |  |  | *LL* | *UL* |  |
| Fixed effects |  |  |  |  |  |
| Intercept | 0.68 | 0.70 | -0.67 | 2.02 | .337 |
| Sex ^b^ | -2.36 | 1.43 | -5.11 | 0.39 | .105 |
| Social anxiety ^c^ | -0.35 | 1.11 | -2.49 | 1.78 | .751 |
| Agreeableness ^d^ | 0.42 | 0.58 | -0.70 | 1.54 | .477 |
| Congruence ^e^ | 1.26 | 0.37 | 0.53 | 2.00 | < .001 |
| Social anxiety × congruence | 1.66 | 0.69 | 0.30 | 3.02 | .017 |
| Agreeableness × congruence | 0.17 | 0.37 | -0.56 | 0.90 | .643 |
| Random effects |  |  |  |  |  |
| Within-subject variance | 16.61 |  | 10.26 | 23.62 |  |
| Within-stimulus variance | 0.97 |  | 0.20 | 2.42 |  |

*Note.* Number of subjects = 57, number of stimuli = 33, total *N* = 5,674. CI = confidence interval; *LL* = lower limit; *UL* = upper limit.

*^a^* (Saucier, 1994) ^b^ -0.5 = male, 0.5 = female. ^c^ Social anxiety was centered within each task. ^d^ Agreeableness was centered within each task. ^e^ 0.5 = incongruent, 0.5 = congruent.

**Table S10.**

*Exploratory analysis controlling for Neuroticism subscale of the Big-Five personality trait ^a^*

| Effect | Estimate | *SE* | 95% CI | | *p* |
| --- | --- | --- | --- | --- | --- |
|  |  |  | *LL* | *UL* |  |
| Fixed effects |  |  |  |  |  |
| Intercept | 0.82 | 0.69 | -0.51 | 2.15 | .242 |
| Sex ^b^ | -2.92 | 1.45 | -5.71 | -0.13 | .049 |
| Social anxiety ^c^ | 0.88 | 1.34 | -1.69 | 3.45 | .513 |
| Neuroticism ^d^ | -0.98 | 0.61 | -2.14 | 0.19 | .113 |
| Congruence ^e^ | 1.26 | 0.37 | 0.53 | 2.00 | < .001 |
| Social anxiety × congruence | 1.75 | 0.80 | 0.17 | 3.32 | .029 |
| Neuroticism × congruence | -0.06 | 0.38 | -0.80 | 0.67 | .867 |
| Random effects |  |  |  |  |  |
| Within-subject variance | 15.90 |  | 9.79 | 22.64 |  |
| Within-stimulus variance | 0.97 |  | 0.20 | 2.42 |  |

*Note.* Number of subjects = 57, number of stimuli = 33, total *N* = 5,674. CI = confidence interval; *LL* = lower limit; *UL* = upper limit.

*^a^* (Saucier, 1994) ^b^ -0.5 = male, 0.5 = female. ^c^ Social anxiety was centered within each task. ^d^ Neuroticism was centered within each task. ^e^ 0.5 = incongruent, 0.5 = congruent.

**Table S11.**

*Exploratory analysis predicting N400 of correctly answered trials at midline and lateral centroparietal sites as a function of social anxiety, congruence, and task.*

| Effect | Estimate | *SE* | 95% CI | | *p* |
| --- | --- | --- | --- | --- | --- |
|  |  |  | *LL* | *UL* |  |
| Fixed effects |  |  |  |  |  |
| Intercept | 0.22 | 0.34 | -0.44 | 0.88 | .522 |
| Sex ^a^ | -0.94 | 0.70 | -2.30 | 0.42 | .185 |
| Social anxiety ^b^ | 0.50 | 0.62 | -0.69 | 1.70 | .419 |
| Congruence ^c^ | 0.96 | 0.25 | 0.48 | 1.44 | < .001 |
| Task ^d^ | 0.33 | 0.69 | -1.00 | 1.66 | .629 |
| Social anxiety × congruence | 0.48 | 0.46 | -0.43 | 1.39 | .298 |
| Social anxiety × task | -0.72 | 1.22 | -3.08 | 1.63 | .555 |
| Congruence × task | 0.62 | 0.49 | -0.34 | 1.59 | .206 |
| Social anxiety × congruence × task | 2.32 | 0.93 | 0.50 | 4.13 | .013 |
| Random effects |  |  |  |  |  |
| Within-subject variance | 9.35 |  | 6.48 | 12.24 |  |
| Within-stimulus variance | 0.47 |  | 0.05 | 1.10 |  |

*Note.* Number of subjects = 107, number of stimuli = 66, total *N* = 11,032. CI = confidence interval; *LL* = lower limit; *UL* = upper limit. Midline and lateral centroparietal sites included Cz, C3, C4, CPz, CP1, CP2, CP5, CP6, Pz, P3, P4, P7, and P8 electrodes.

^a^ -0.5 = male, 0.5 = female. ^b^ Social anxiety was centered within each task. ^c^ -0.5 = incongruent, 0.5 = congruent. ^d^ 0.5 = RME, -0.5 = Control.

**Figure S1.**

*Figure S1*. The grand averaged N400 waveforms from midline and lateral centroparietal electrodes (i.e., averaged from Cz, C3, C4, CPz, CP1, CP2, CP5, CP6, Pz, P3, P4, P7, and P8) in the (A) RME task and (B) Control task. The relationship between midline and lateral centroparietal N400 and social anxiety in the (C) RME task and (D) Control task. This relationship is only significant in the RME task. Error ribbons indicate standard error.

**Figure S2.**

*Figure S2*. The scalp topography of the LPP mean amplitude. Panels (A) and (B) show the LPP for congruent and incongruent trials in the RME task, respectively. Panels (C) and (D) show the LPP for congruent and incongruent trials in the Control task, respectively. Scale is in μV and ranges from red (higher positivity) to blue (lower positivity). As in prior work (Cuthbert et al., 2000), the LPP is pronounced across the centroparietal sites. The LPP was analyzed by averaging the ERPs at the midline electrodes (Cz, CPz, and Pz) and across an extended centroparietal cluster including lateral sites.

**Table S12.**

*Exploratory analysis predicting Late Positive Potential (600-1000ms) of correctly answered trials at midline and lateral centroparietal sites as a function of social anxiety, congruence, and task.*

| Effect | Estimate | *SE* | 95% CI | | *p* |
| --- | --- | --- | --- | --- | --- |
|  |  |  | *LL* | *UL* |  |
| Fixed effects |  |  |  |  |  |
| Intercept | 0.16 | 0.38 | -0.58 | 0.90 | .673 |
| Sex ^a^ | 0.27 | 0.79 | -1.27 | 1.80 | .738 |
| Social anxiety ^b^ | 0.37 | 0.70 | -0.97 | 1.72 | .593 |
| Congruence ^c^ | 1.81 | 0.28 | 1.26 | 2.36 | < .001 |
| Task ^d^ | 0.03 | 0.77 | -1.45 | 1.51 | .970 |
| Social anxiety × congruence | 0.75 | 0.53 | -0.29 | 1.78 | .158 |
| Social anxiety × task | 0.21 | 1.37 | -2.44 | 2.86 | .881 |
| Congruence × task | 0.54 | 0.56 | -0.56 | 1.64 | .338 |
| Social anxiety × congruence × task | 1.26 | 1.06 | -0.81 | 3.33 | .234 |
| Random effects |  |  |  |  |  |
| Within-subject variance | 11.81 |  | 8.18 | 15.46 |  |
| Within-stimulus variance | 0.37 |  | 0.00 | 1.05 |  |

*Note.* Number of subjects = 107, number of stimuli = 66, total *N* = 11,032. CI = confidence interval; *LL* = lower limit; *UL* = upper limit. Midline and lateral centroparietal sites included Cz, C3, C4, CPz, CP1, CP2, CP5, CP6, Pz, P3, P4, P7, and P8 electrodes.

^a^ -0.5 = male, 0.5 = female. ^b^ Social anxiety was centered within each task. ^c^ -0.5 = incongruent, 0.5 = congruent. ^d^ 0.5 = RME, -0.5 = Control.

**Figure S3.**

*Figure S3*. The relationship between social anxiety and centroparietal LPP. Panels (A) and (B) show midline LPP in the RME and Control tasks, respectively. Panels (C) and (D) show midline and lateral LPP in the RME and Control tasks, respectively. The relationship is marginal in the RME task. The social anxiety × congruence × task three-way interaction did not reach statistical significance. Error ribbons indicate standard error.

**Table S13.**

*Exploratory analysis predicting N400 of correctly answered trials at midline centroparietal sites using adjusted peak-latency time windows as a function of social anxiety, congruence, and task.*

| Effect | Estimate | *SE* | 95% CI | | *p* |
| --- | --- | --- | --- | --- | --- |
|  |  |  | *LL* | *UL* |  |
| Fixed effects |  |  |  |  |  |
| Intercept | 0.38 | 0.43 | -0.44 | 1.21 | .374 |
| Sex ^a^ | -1.98 | 0.88 | -3.69 | -0.28 | .026 |
| Social anxiety ^b^ | 0.85 | 0.77 | -0.64 | 2.35 | .273 |
| Congruence ^c^ | 1.18 | 0.28 | 0.64 | 1.72 | < .001 |
| Task ^d^ | 0.65 | 0.86 | -1.01 | 2.30 | .452 |
| Social anxiety × congruence | 0.53 | 0.52 | -0.50 | 1.55 | .313 |
| Social anxiety × task | -1.18 | 1.52 | -4.13 | 1.76 | .439 |
| Congruence × task | 0.61 | 0.55 | -0.47 | 1.70 | .268 |
| Social anxiety × congruence × task | 2.81 | 1.04 | 0.77 | 4.85 | .007 |
| Random effects |  |  |  |  |  |
| Within-subject variance | 15.11 |  | 10.62 | 19.63 |  |
| Within-stimulus variance | 0.62 |  | 0.09 | 1.43 |  |

*Note.* Number of subjects = 107, number of stimuli = 66, total *N* = 11,032. CI = confidence interval; *LL* = lower limit; *UL* = upper limit. Midline centroparietal sites included Cz, CPz, and Pz electrodes. N400 amplitudes were extracted using adjusted peak-latency time windows defined separately for each Task × Social Anxiety (High / Low; median split) subgroup.

^a^ -0.5 = male, 0.5 = female. ^b^ Social anxiety was centered within each task. ^c^ -0.5 = incongruent, 0.5 = congruent. ^d^ 0.5 = RME, -0.5 = Control.

**Table S14.**

*Exploratory analysis predicting N400 of correctly answered trials at midline and lateral centroparietal sites using adjusted peak-latency time windows as a function of social anxiety, congruence, and task.*

| Effect | Estimate | *SE* | 95% CI | | *p* |
| --- | --- | --- | --- | --- | --- |
|  |  |  | *LL* | *UL* |  |
| Fixed effects |  |  |  |  |  |
| Intercept | 0.21 | 0.34 | -0.45 | 0.87 | .540 |
| Sex ^a^ | -0.86 | 0.70 | -2.21 | 0.49 | .223 |
| Social anxiety ^b^ | 0.54 | 0.62 | -0.66 | 1.73 | .387 |
| Congruence ^c^ | 1.00 | 0.24 | 0.52 | 1.48 | < .001 |
| Task ^d^ | 0.31 | 0.68 | -1.01 | 1.63 | .650 |
| Social anxiety × congruence | 0.47 | 0.46 | -0.43 | 1.37 | .307 |
| Social anxiety × task | -0.85 | 1.21 | -3.19 | 1.49 | .485 |
| Congruence × task | 0.56 | 0.49 | -3.19 | 1.49 | .252 |
| Social anxiety × congruence × task | 2.39 | 0.92 | 0.58 | 4.19 | .001 |
| Random effects |  |  |  |  |  |
| Within-subject variance | 9.26 |  | 6.41 | 12.12 |  |
| Within-stimulus variance | 0.46 |  | 0.05 | 1.08 |  |

*Note.* Number of subjects = 107, number of stimuli = 66, total *N* = 11,032. CI = confidence interval; *LL* = lower limit; *UL* = upper limit. Midline centroparietal sites included Cz, C3, C4, CPz, CP1, CP2, CP5, CP6, Pz, P3, P4, P7, and P8 electrodes. N400 amplitudes were extracted using adjusted peak-latency time windows defined separately for each Task × Social Anxiety (High / Low; median split) subgroup.

^a^ -0.5 = male, 0.5 = female. ^b^ Social anxiety was centered within each task. ^c^ -0.5 = incongruent, 0.5 = congruent. ^d^ 0.5 = RME, -0.5 = Control.

**References**

Hezel, D. M., & McNally, R. J. (2014). Theory of mind impairments in social anxiety disorder. *Behavior Therapy*, *45*(4), 530–540. https://doi.org/10.1016/j.beth.2014.02.010

Saucier, G. (1994). Mini-Markers: A Brief Version of Goldberg’s Unipolar Big-Five Markers. *Journal of Personality Assessment*, *63*(3), 506–216. https://doi.org/10.1207/s15327752jpa6303

Singelis, T. M. (1994). The measurement of independent and interdependent self-construals. *Personality and Social Psychology Bulletin*, *20*(5), 580–591.
